# Supplementary material for: Quorum sensing signals of the grapevine crown gall bacterium, Novosphingobium sp. Rr2-17: use of inducible expression and polymeric resin to sequester acyl-homoserine lactones
Source: PeerJ. 2024 Dec 20;12:e18657. doi: 10.7717/peerj.18657 (PMC11674143; doi:10.7717/peerj.18657)
Supplement: Supplemental Information 1 — Alignment of NovISpR1 (green arrow) and other putative Novosphingobium LuxI homologs with the functionally confirmed LuxI homologs from Rhizobium radiobacter and Agrobacterium fabrum (lines 1 and 2, above green arrow). Highly conserved amino acid residues were highlighted. [file peerj-12-18657-s001.pdf]

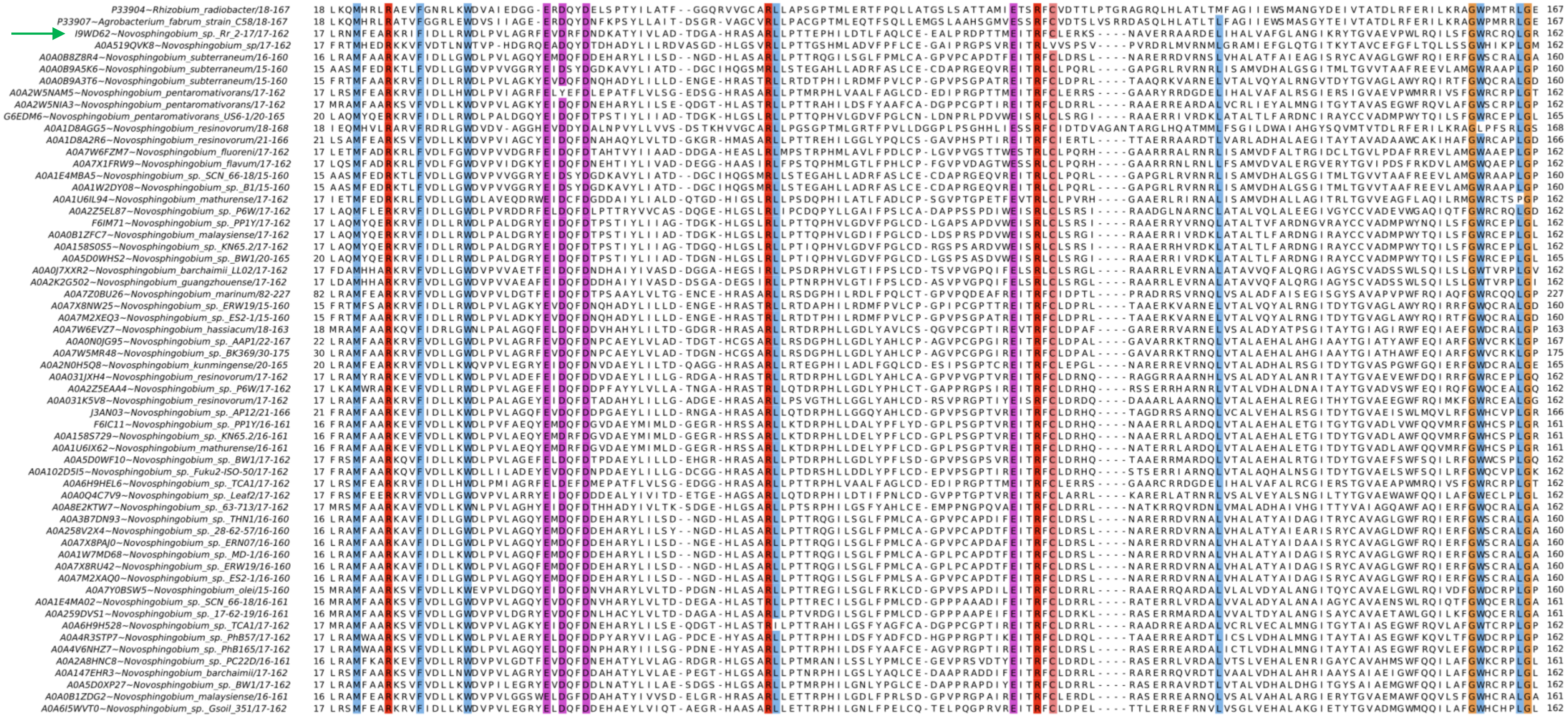

P33904~Rhizobium_radiobacter/18-167
P33907~Agrobacterium_fabrum_strain_C58/18-167
I9WD62~Novosphingobium_sp._Rr_2-17/17-162
A0A519QVK8~Novosphingobium_sp/17-162
A0A0B8Z8R4~Novosphingobium_subterraneum/16-160
A0A0B9A5K6~Novosphingobium_subterraneum/15-160
A0A0B9A3T6~Novosphingobium_subterraneum/15-160
A0A2W5NAM5~Novosphingobium_pentaromativorans/17-162
A0A2W5NIA3~Novosphingobium_pentaromativorans/17-162
G6EDM6~Novosphingobium_pentaromativorans_US6-1/20-165
A0A1D8AGG5~Novosphingobium_resinovorum/18-168
A0A1D8A2R6~Novosphingobium_resinovorum/21-166
A0A7W6FZM7~Novosphingobium_fluoreni/17-162
A0A7X1FRW9~Novosphingobium_flavum/17-162
A0A1E4MBA5~Novosphingobium_sp._SCN_66-18/15-160
A0A1W2DY08~Novosphingobium_sp._B1/15-160
A0A1U6IL94~Novosphingobium_mathurense/17-162
A0A2Z5EL87~Novosphingobium_sp._P6W/17-162
F6IM71~Novosphingobium_sp._PP1Y/17-162
A0A0B1ZFC7~Novosphingobium_malaysiense/17-162
A0A158S0S5~Novosphingobium_sp._KN65.2/17-162
A0A5D0WHS2~Novosphingobium_sp._BW1/20-165
A0A0J7XXR2~Novosphingobium_barchaimii_LL02/17-162
A0A2K2G502~Novosphingobium_guangzhouense/17-162
A0A7Z0BU26~Novosphingobium_marinum/82-227
A0A7X8NW25~Novosphingobium_sp._ERW19/15-160
A0A7M2XEQ3~Novosphingobium_sp._ES2-1/15-160
A0A7W6EVZ7~Novosphingobium_hassiacum/18-163
A0A0N0JG95~Novosphingobium_sp._AAP1/22-167
A0A7W5MR48~Novosphingobium_sp._BK369/30-175
A0A2N0H5Q8~Novosphingobium_kunmingense/20-165
A0A031JXH4~Novosphingobium_resinovorum/17-162
A0A2Z5EAA4~Novosphingobium_sp._P6W/17-162
A0A031K5V8~Novosphingobium_resinovorum/17-162
J3AN03~Novosphingobium_sp._AP12/21-166
F6IC11~Novosphingobium_sp._PP1Y/16-161
A0A158S729~Novosphingobium_sp._KN65.2/16-161
A0A1U6IX62~Novosphingobium_mathurense/16-161
A0A5D0WF10~Novosphingobium_sp._BW1/17-162
A0A102D5I5~Novosphingobium_sp._Fuku2-ISO-50/17-162
A0A6H9HEL6~Novosphingobium_sp._TCA1/17-162
A0A0Q4C7V9~Novosphingobium_sp._Leaf2/17-162
A0A8E2KTW7~Novosphingobium_sp._63-713/17-162
A0A3B7DN93~Novosphingobium_sp._THN1/16-160
A0A258V2X4~Novosphingobium_sp._28-62-57/16-160
A0A7X8PAJ0~Novosphingobium_sp._ERN07/16-160
A0A1W7MD68~Novosphingobium_sp._MD-1/16-160
A0A7X8RU42~Novosphingobium_sp._ERW19/16-160
A0A7M2XAQ0~Novosphingobium_sp._ES2-1/16-160
A0A7Y0BSW5~Novosphingobium_olei/15-160
A0A1E4MA02~Novosphingobium_sp._SCN_66-18/16-161
A0A259DVS1~Novosphingobium_sp._17-62-19/16-161
A0A6H9H528~Novosphingobium_sp._TCA1/17-162
A0A4R3STP7~Novosphingobium_sp._PhB57/17-162
A0A4V6NHZ7~Novosphingobium_sp._PhB165/17-162
A0A2A8HNC8~Novosphingobium_sp._PC22D/16-161
A0A147EHR3~Novosphingobium_barchaimii/17-162
A0A5D0XP27~Novosphingobium_sp._BW1/17-162
A0A0B1ZDG2~Novosphingobium_malaysiense/16-161
A0A6I5WVT0~Novosphingobium_sp._Gsoil_351/17-162
