# Supplementary figures and images for: Quorum sensing signals of the grapevine crown gall bacterium, Novosphingobium sp. Rr2-17: use of inducible expression and polymeric resin to sequester acyl-homoserine lactones
Source: PeerJ. 2024 Dec 20;12:e18657. doi: 10.7717/peerj.18657 (PMC11674143; doi:10.7717/peerj.18657)

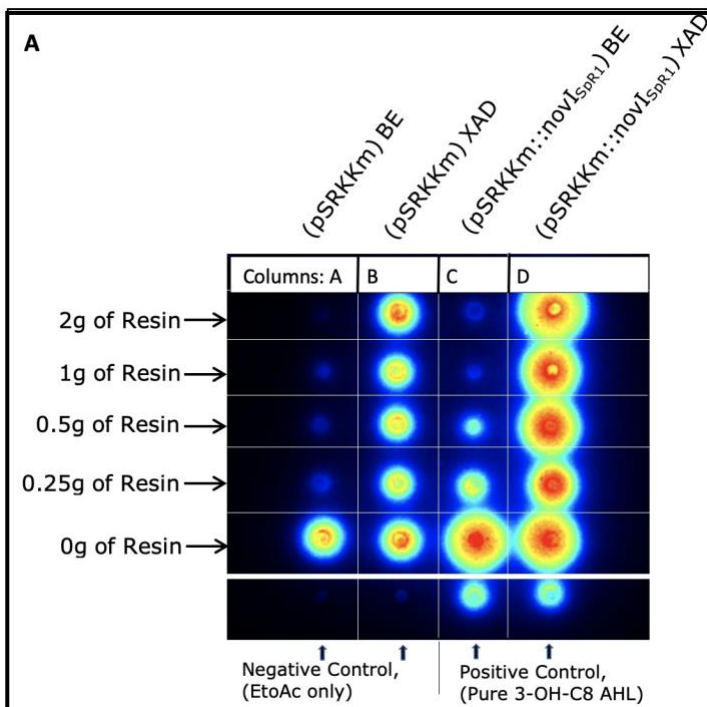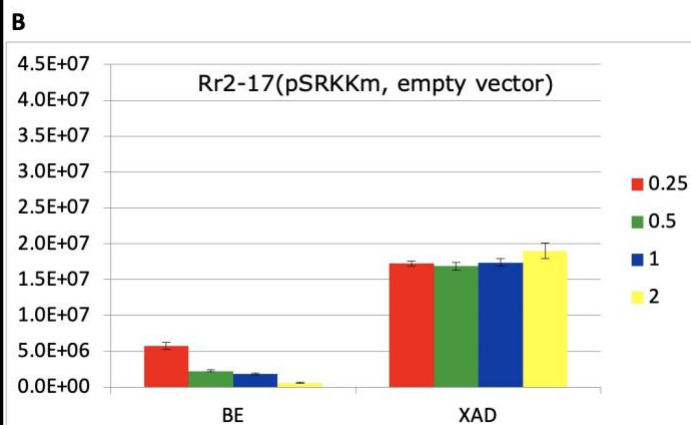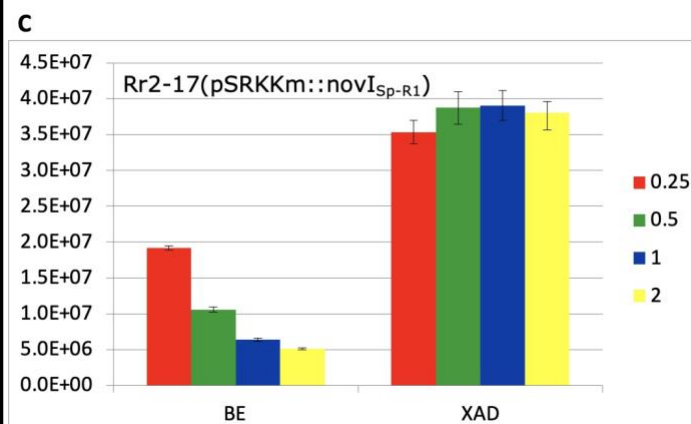

Supplement: Supplemental Information 2 — (A) Image was captured using Bio-Rad ChemiDoc Mp Imaging System with an exposure time of 35 seconds. Bioluminescent responses are shown of total AHLs with increasing concentrations of XAD resin (XAD) extracts and broth (BE) extracts from broth media cultures of Rr2-17 (pSRKKm) under IPTG inducible conditions and the same from broth media cultures of Rr2-17 (pSRKKm::novISp-R1) under IPTG inducible conditions. (B) Bioluminescent quantification of total AHLs extracted from resin and culture from broth cultures containing different concentrations of resin (2.0. 1.0, 0.5, 0.25 %) of Rr2-17 (pSRKKm) under IPTG inducible conditions and (C) the same of cultures of Rr2-17 (pSRKKm::novIspR1). Abbreviations: Broth extracted, BE; Resin XAD-16 extracted, XAD. [file peerj-12-18657-s002.pdf]

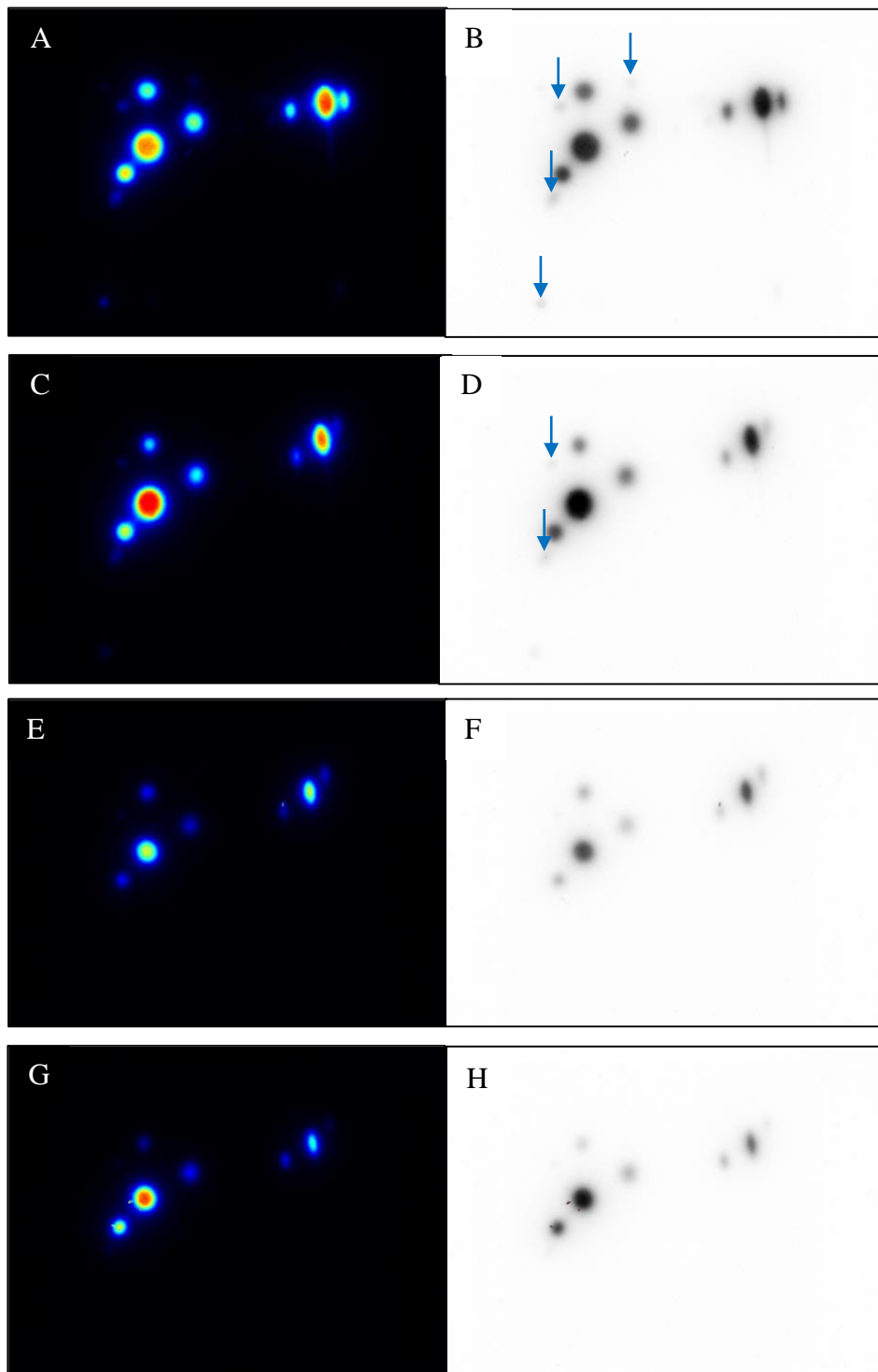

Supplement: Supplemental Information 4 — 2-D TLC of the Rr2-17(pSRKKm::novIspR1) resin extract (A, B) and culture extract (C, D) after imaging with the bioluminescent biosensor A136 in color (A, C) and black & white (B&W) detection (B, D). 2-D TLC of the Rr2-17 (pSRKKm) resin extract (E, F) and culture extract (G, H) after imaging with the bioluminescent biosensor A136 in color (E, G) and B&W detection (F, H). Blue arrows indicate four and two signal spots observed in panels A, B and C, D, respectively, that are not visible in resin or culture extracts from Rr2-17 (pSRKKm) (E, F and G, H), respectively. Origin of extract is as noted in figure 6. [file peerj-12-18657-s004.pdf]
