# Supplementary material for: Quorum sensing signals of the grapevine crown gall bacterium, Novosphingobium sp. Rr2-17: use of inducible expression and polymeric resin to sequester acyl-homoserine lactones
Source: PeerJ. 2024 Dec 20;12:e18657. doi: 10.7717/peerj.18657 (PMC11674143; doi:10.7717/peerj.18657)
Supplement: Supplemental Information 3 [file peerj-12-18657-s003.pdf]

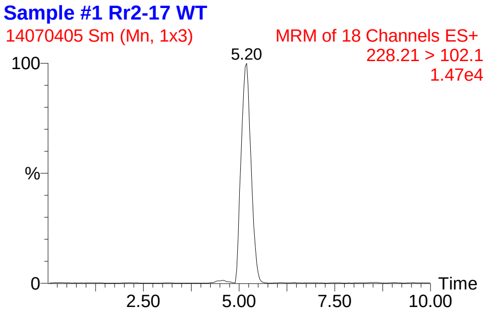

C8

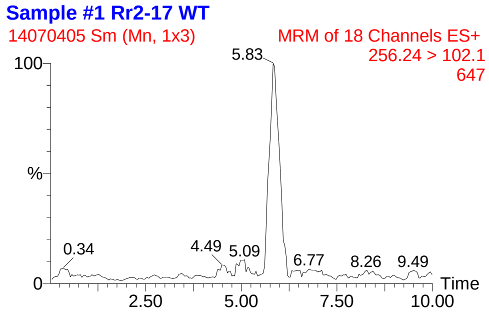

C10

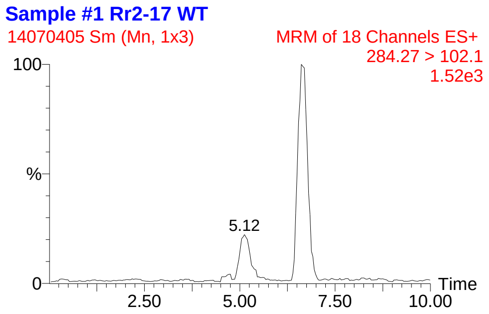

C12

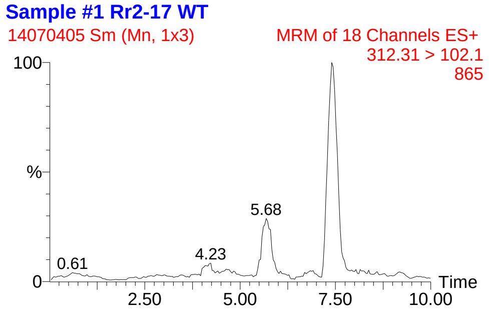

C14

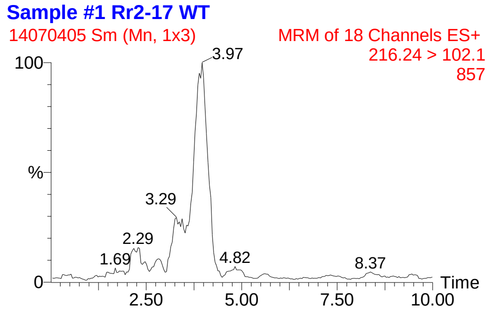

3-OH-C6

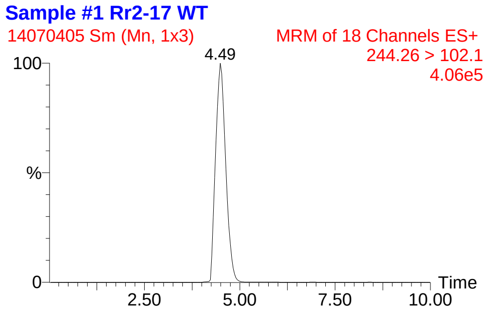

3-OH-C8

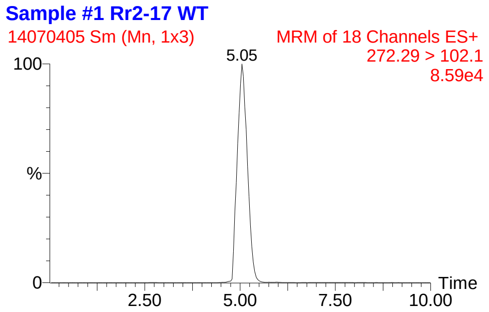

3-OH-C10

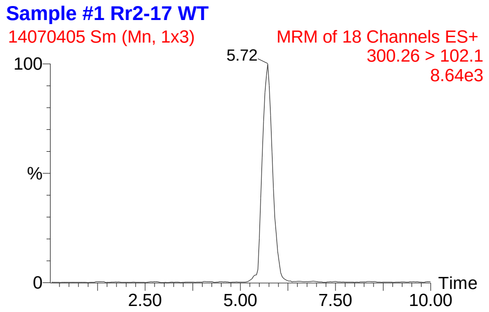

3-OH-C12

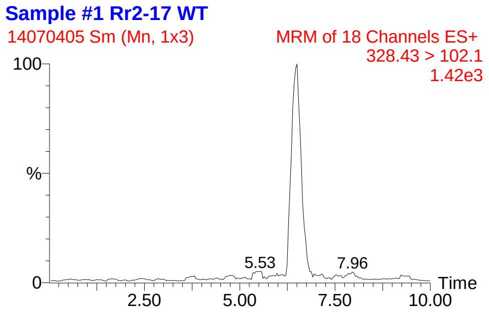

3-OH-C14
