# Supplementary material for: Quorum sensing signals of the grapevine crown gall bacterium, Novosphingobium sp. Rr2-17: use of inducible expression and polymeric resin to sequester acyl-homoserine lactones
Source: PeerJ. 2024 Dec 20;12:e18657. doi: 10.7717/peerj.18657 (PMC11674143; doi:10.7717/peerj.18657)
Supplement: Supplemental Information 5 — 1 C4-HSL, N-butanoyl-homoserine lactone; C6-HSL, N-hexanoyl-homoserine lactone; 3-oxo-C6- HSL, N-3-oxo-hexanoyl-homoserine lactone; 3-oxo-C8-HSL, N-3-oxo-octanoyl-homoserine lactone; 3-oxo-C12-HSL, N-oxo-dodecanoyl-homoserine lactone. [file peerj-12-18657-s005.pdf]

Supplemental Table 1. **Whole cell biosensor strains used in this work.**

**Biosensor strain**	**Receptor**	**Cognate AHL[1]**	**Reporter**
A. tumefaciens A136 (pCF218, pMV26)	TraR	3-oxo-C8-HSL	*luxCDABE*
A. tumefaciens NTL4 (pZLR4)	TraR	3-oxo-C8-HSL	β-galactosidase
C. violaceum CV026	CviR	C6-HSL	Violacein Pigment
E. coli JM109 (pSB401)	LuxR	3-oxo-C6-HSL	*luxCDABE*
E. coli JM109 (pSB536	AhyR	C4-HSL	*luxCDABE*
E. coli JM109 (pSB1075)	LasR	3-oxo-C12-HSL	*luxCDABE*

[1] C4-HSL, *N*-butanoyl-homoserine lactone; C6-HSL, *N*-hexanoyl-homoserine lactone; 3-oxo-C6-HSL, *N*-3-oxo-hexanoyl-homoserine lactone; 3-oxo-C8-HSL, *N*-3-oxo-octanoyl-homoserine lactone; 3-oxo-C12-HSL, *N*-oxo-dodecanoyl-homoserine lactone.
