# Supplementary material for: Quorum sensing signals of the grapevine crown gall bacterium, Novosphingobium sp. Rr2-17: use of inducible expression and polymeric resin to sequester acyl-homoserine lactones
Source: PeerJ. 2024 Dec 20;12:e18657. doi: 10.7717/peerj.18657 (PMC11674143; doi:10.7717/peerj.18657)
Supplement: Supplemental Information 6 [file peerj-12-18657-s006.pdf]

Supplemental Table 2. **Mass transitions used for the MRM detection of common AHLs.**

**Acyl chain length**	**Carbon 3 substitution**	**MRMs**	**Retention time / min**
C4	Unsubstituted	172-102	3.18
	Oxo	186-102	2.02
	OH	188-102	1.58
C6	Unsubstituted	200-102	4.53
	Oxo	214-102	4.08
	OH	216-102	3.93
C8	Unsubstituted	228-102	5.20
	Oxo	242-102	4.66
	OH	244-102	4.49
C10	Unsubstituted	256-102	5.87
	Oxo	270-102	5.31
	OH	272-102	5.05
C12	Unsubstituted	284-102	6.65
	Oxo	298-102	5.98
	OH	300-102	5.72
C14	Unsubstituted	312-102	7.44
	Oxo	326-102	6.77
	OH	328-102	6.50
