# Supplementary material for: Quorum sensing signals of the grapevine crown gall bacterium, Novosphingobium sp. Rr2-17: use of inducible expression and polymeric resin to sequester acyl-homoserine lactones
Source: PeerJ. 2024 Dec 20;12:e18657. doi: 10.7717/peerj.18657 (PMC11674143; doi:10.7717/peerj.18657)
Supplement: Supplemental Information 7 [file peerj-12-18657-s007.pdf]

Supplemental Table 3. **Genome statistics of *Novosphingobium* sp. Rr2-17.**

Strain	A.N.	Size (bp)	#contigs	N50	GC%	Characteristics	Reference
N. sp. Rr2-17	AKFJ01000000	4,539,029	166	130,074	62.7%	Nopaline-type grapevine tumor isolate, Hungary	Gan et al., 2009
