# Supplementary material for: Quorum sensing signals of the grapevine crown gall bacterium, Novosphingobium sp. Rr2-17: use of inducible expression and polymeric resin to sequester acyl-homoserine lactones
Source: PeerJ. 2024 Dec 20;12:e18657. doi: 10.7717/peerj.18657 (PMC11674143; doi:10.7717/peerj.18657)
Supplement: Supplemental Information 8 [file peerj-12-18657-s008.pdf]

Supplementary Table 4. **Secondary metabolite biosynthetic gene clusters identified by antiSMASH platform.**

Strain	Cluster	Type	Genes showing homology	Contig#	Location
N. sp. Rr2-17	1	Type III polyketides		17	101702-142751
	2	Terpene: Astaxanthin dideoxyglycoside	50%	91	3435-35543
	3	Homoserine lactone		97	3765-24379
	4	Siderophore		153	1-9588
