# Supplementary material for: Quorum sensing signals of the grapevine crown gall bacterium, Novosphingobium sp. Rr2-17: use of inducible expression and polymeric resin to sequester acyl-homoserine lactones
Source: PeerJ. 2024 Dec 20;12:e18657. doi: 10.7717/peerj.18657 (PMC11674143; doi:10.7717/peerj.18657)
Supplement: Supplemental Information 9 [file peerj-12-18657-s009.pdf]

Supplementary Table 5. **LC-MS/MS quantification of three acyl homoserine lactones in μM in resin (XAD) and residual broth culture (BE) and from broth culture without resin (BC).**

Strain, plasmid, resin extract (XAD), broth extract (BE) fractions or broth culture only control (BC)	AHL Signal in μM		
	C8	OH-C8	OH-C10
Rr2-17 (pSRKKm) - 0.25% XAD	0.04	2.25	0.21
Rr2-17 (pSRKKm) - 0.5% XAD	0.06	2.91	0.28
Rr2-17 (pSRKKm) - 1.0% XAD	0.04	2.52	0.22
Rr2-17 (pSRKKm) - 2.0% XAD	0.07	2.87	0.36
Rr2-17 (pSRKKm) - 0.25% BE	nd	0.14	nd
Rr2-17 (pSRKKm) - 0.5% BE	nd	0.06	nd
Rr2-17 (pSRKKm) - 1.0% BE	nd	0.03	nd
Rr2-17 (pSRKKm) - 2.0% BE	nd	nd	nd
Rr2-17 (pSRKKm) - BC control	0.05	1.56	0.27
Rr2-17 (pSRKKm::novIspR1) - 0.25% XAD	0.23	15.81	1.63
Rr2-17 (pSRKKm::novIspR1) - 0.5% XAD	0.3	17.52	1.95
Rr2-17 (pSRKKm::novIspR1) - 1.0% XAD	0.28	18.28	1.84
Rr2-17 (pSRKKm::novIspR1) - 2.0% XAD	0.58	19.48	3.33
Rr2-17 (pSRKKm::novIspR1) - 0.25% BE	nd	1.28	nd
Rr2-17 (pSRKKm::novIspR1) - 0.5% BE	nd	0.61	nd
Rr2-17 (pSRKKm::novIspR1) - 1.0% BE	nd	0.15	nd
Rr2-17 (pSRKKm::novIspR1) - 2.0% BE	nd	0.14	nd
Rr2-17 (pSRKKm::novIspR1) - BC control	0.25	11.05	1.93
